# Subdural haematoma presenting as catatonia in a 20-year-old male: a case report

**DOI:** 10.4076/1757-1626-2-8032

**Published:** 2009-09-03

**Authors:** Bawo Onesirosan James, Joyce Ohiole Omoaregba, Ambrose Onivefu Lawani, Charles Onyenibedi Ikeji, Nosa Godwin Igbinowanhia

**Affiliations:** 1 Department of Clinical Services, Federal Neuropsychiatric Hospital, Benin-City, Edo State, Nigeria

## Abstract

**Introduction:**

Catatonia is a common presentation to psychiatric services in developing countries. Medical causes of catatonia are common and often missed. Identifying causes for catatonia is important not only to guide proper management but to determine prognostic outcomes

**Case presentation:**

We report a case of a 20-year-old male who presented with catatonia. Subsequent investigations revealed a chronic subdural haematoma. Implications of late presentation to orthodox services are discussed.

**Conclusion:**

Careful clinical observation, investigation and a high index of suspicion are necessary to effectively manage this condition.

## Introduction

Subdural haematomas in its' acute or chronic form is a recognized cause of catatonia [1]-[3]. Subdural haematomas secondary to traumatic brain injuries though common in the elderly may also occur in young or middle aged persons [4]. A retrospective case review showed that catatonic patients were significantly more likely to have had a prior history of brain injury when compared to manic, depressed or surgical patients used as controls. The study suggested a possible association between catatonia and brain dysfunction [5]. Recently, a case report highlighted the clinical and treatment implications of using neuroleptics on brain injured persons with catatonic symptoms [6]. Though, there is as yet no consensus on managing catatonia in persons with brain injuries, clozapine and risperidone have been found to be effective in some case reports [7,8].

Catatonia is still largely under-diagnosed and under-recognised [9], and medical catatonia (catatonia with a medical co-morbidity) which accounts for 30-80% of all catatonias [4] is often missed by health professionals. Though catatonia is reported to be on the decline in developed countries, this is not the case in developing nations, where delay in seeking psychiatric treatment and higher frequency of organic causes of mental illness still persist and are believed to account for a higher prevalence[10]. We report the case of a patient presenting with catatonic symptoms without obvious neurological deficits, for which subsequent brain imaging revealed a chronic subdural haematoma.

## Case presentation

A 20-year-old Nigerian male, of Ibo ethnic origin presented with a 5 year history of irrational speech, irritability and refusal to do house chores. Three months after the onset of his symptoms, he became very prayerful, expressed grandiose ideas of being a priest with a special call from God and chose to spend a significant part of his weekdays at his local church, alone, reading the bible. Over the next two years he had episodes of social withdrawal, fatuous laughter and 2^nd^ person auditory hallucinations which had completely remitted at the time of his presentation. It was noticed about a year ago that he would remain mute for extended periods of time (mutism), maintain a particular position e.g. standing and gazing upwards for hours (posturing), would perform the opposing action of an instruction or task given by his parents (negativism), repeats questions asked or statements made to him severally (echolalia), and would often perform repetitive apparently goal directed hand-washing and writing movements (mannerisms).

In addition to being managed for febrile convulsions at age 2 and severe malaria (plasmodiases) with anaemia at age 7, he sustained a head injury at a saw mill at age 13 where he worked as a hired hand. The injury involved a plank which fell from an unspecified height with the result that he lost consciousness for about thirty minutes. The resulting scalp laceration was sutured at a primary health care facility. After he regained consciousness, no further investigations were conducted at the time. He had been treated at two traditional/faith-based (religious) centres since the onset of behavioural symptoms with herbal medications and prayers which did not improve his symptomatology. There was no prior psychiatric history.

He suffered pre-natal complications from a pre-term rupture of membranes that required an emergency caesarean section at his delivery, however developmental milestones were within normal limits and his early childhood and education up to junior secondary school were satisfactory. There was a gradual decline in academic performance following the incident (injury to the head) and he stopped schooling altogether five years ago after the onset of symptoms.

Mental status examination revealed an asthenic, fairly groomed young male in appropriate attire with poor eye contact. He was alert and oriented (responded to statements eliciting time, place and person by nodding his head). He remained mute and did not reply verbally to questions or instructions throughout the examination. He was rigid, exhibited posturing, mannerisms and ambitendence.

On physical examination, he was, slightly pale with systemic examination being essentially normal and within normal limits. A neurological examination, showed no evidence of lateralizing signs, muscle tone, power and reflexes were normal.

Laboratory tests, including chemistry (electrolytes and urea) and urinalysis were within normal limits. A complete blood count only revealed a mild anaemia (PCV: 24%). A cranial Computerised Tomography (CT-Scan) was also requested and revealed a frontal bilaterally located, concavo-convex chronic subdural haematoma with associated dilatation of the anterior pole of the lateral ventricles.

In patient admission was declined at the first interview due to financial constraints. For similar reasons a cranial CT scan that was requested could not be performed until the third week after presentation (CT scans on the average costs $200 - $300 in this part of the country). An initial trial of oral lorazepam at 4 mg in two divided doses showed minimal response with only a slight improvement in speech after the first week. A course of 6 sessions of electroconvulsive therapy (ECT) subsequently prescribed before the CT-Scan was done, was discontinued after the third session due to poor response and marked post-ECT confusion. Efforts were geared toward performing a cranial CT scan to guide further intervention as an organic aetiology was now highly considered. Following the outcome of the CT scan, a neurosurgical consult was requested and they advised a continuation and/or exploration of conservative management as there were no indications for surgery. Also a re-examination of the CT-scan suggested that areas of white brain matter were visible within the area of the chronic subdural haematoma implying that an active re-absorption of the collected blood was ongoing, moreover the absence of any lateralizing signs suggested that intracranial compensatory mechanisms were likely occurring.

We commenced a trial of low dose risperidone at 1 mg nocte over two weeks, with subsequent 1 mg dosage increase 4 weekly. Two months later, at a dose of 3 mg nocte, the parents noted that they had observed remarkable improvement. They reported that he could now initiate conversations, appeared interested in his environment, requested for meals and maintained a good personal hygiene with little persuasion. However, they also reported that he still had episodes of posturing, though they were reduced in terms of the duration that it occurred. Efforts to repeat the CT scan was unsuccessful as the care givers complained of inability to foot the bill.

## Discussion

Catatonia is a rare presenting cluster of symptoms for subdural haematoma. The CT scan finding of a chronic subdural haematoma raises some questions. First, the long duration from time of head injury to onset of neuropsychiatric symptoms suggests that the likely picture was that of an initial small subdural haemorrhage with recurrent acute-on-chronic bleeds over the years. Evidence for this might also be seen from the effect on the frontal lobe with the result that cerebral atrophy in the frontal lobe region had led to an enlargement of the anterior horns of the lateral ventricles. Also, the young age of the patient and an absence of florid neurological signs or symptoms explain why he did not present to neurological (medical) services.

Poor knowledge of neurological, mental or behavioural disorders among individuals in Nigeria often means that they ascribe these symptoms as having Magico-religious aetiology [11]. Several studies have shown that pathways to care involve visits to spiritual, faith-religious healers and traditional/native health practitioners [11]. This case illustrates the negative effect of these attitudes toward psychiatric/neurological disorders, as late presentation for medical intervention has adverse implications for prognosis and eventual treatment outcome in this case, which is likely to be poor. Also, healthcare services are still financed largely from out-of-pocket expenses [12]. The nation's health insurance scheme is still in its infancy and caters mostly for civil servants that comprise a minority of the populace that need access to healthcare. Failure of the medical team to conduct full imaging investigations at the time of head injury seven years prior to presentation was due not only to the financial constraints of the parents, but the often wrongly held opinion by some general practitioners that cases of head injury without or a short duration of loss of consciousness may not require 'expensive' investigations to rule out subtle or latent brain lesions.

Though antipsychotics are not routinely recommended in the treatment of catatonia, especially of medical origin, Hesslinger and his colleagues [8] reported that a patient with catatonia unresponsive to benzodiazepines showed dramatic and persistent improvement on risperidone. The moderate response to risperidone as observed in this case probably suggests a differential diagnosis of organic delusional (schizophrenia-like) disorder. However, as effective communication with the patient was difficult, this differential could not be explored further.

## Conclusion

Lesions involving the central nervous system should be borne in mind when investigating and managing catatonia. If overt neurological symptoms do not point to a neurosurgical cause, failure to respond to a trial of benzodiazepenes or electroconvulsive therapy should inform a complete investigation of the central nervous system. This recommendation is paramount especially in resource-poor settings where there is often an unwillingness to refer patients for investigations which are usually expensive and beyond the reach of many. Furthermore, efforts should be geared towards making the nations' health insurance scheme more functional for both the public and private sectors. This will reduce the financial burden arising from out-of-pocket payments.

## Abbreviations

CT: computerised tomography; ECT: electroconvulsive therapy; PCV: packed cell volume.

## Consent

Written informed consent was obtained from the patients' father for publication of this case report. A copy of the written consent is available for review from the journal's Editor-in-Chief.

## Competing interests

The authors declare that they have no competing interests.

## Authors' contributions

BOJ conceived the manuscript. BOJ, JOO, AOL and NGI drafted the initial manuscript. BOJ, AOL and OCI edited the manuscript. All authors read and approved the final draft. BOJ, JOO, AOL, OCI & NGI were directly involved in the assessment and management of the patient.
